# Serum levels of matrix metalloproteinases -1,-2,-3 and -9 in thoracic aortic diseases and acute myocardial ischemia

**DOI:** 10.1186/1749-8090-4-59

**Published:** 2009-11-03

**Authors:** Georgios T Karapanagiotidis, Polychronis Antonitsis, Nicholas Charokopos, Christophoros N Foroulis, Kyriakos Anastasiadis, Efthymia Rouska, Helena Argiriadou, Kyriakos Rammos, Christos Papakonstantinou

**Affiliations:** 1 Department of Thoracic and Cardiovascular Surgery, AHEPA University Hospital, Thessaloniki, Greece

## Abstract

**Background:**

Matrix metalloproteinases (MMPs) constitute a family of zinc-dependent proteases (endopeptidases) whose catalytic action is the degradation of the extracellular matrix components. In addition, they play the major role in the degradation of collagen and in the process of tissue remodeling. The present clinical study investigated blood serum levels of metalloproteinases- 1, -2, -3 and -9 in patients with acute and chronic aortic dissection, thoracic aortic aneurysm and acute myocardial ischemia compared to healthy individuals.

**Methods:**

The blood serum levels of MMP-1, -2, -3 and -9 were calculated in 31 patients with acute aortic dissection, 18 patients with chronic aortic dissection, 18 patients with aortic aneurysm and in 13 patients with acute myocardial ischemia, as well as in 15 healthy individuals who served as the control group. Serum MMP levels were measured by using an ELISA technique.

**Results:**

There were significantly higher levels of MMP-3 in patients with acute myocardial ischemia as compared to acute aortic dissection (17.33 ± 2.03 ng/ml versus 12.92 ± 1.01 ng/ml, p < 0.05). Significantly lower levels of MMP-1 were found in healthy controls compared to all groups of patients (1.1 ± 0.38 ng/ml versus 2.97 ± 0.68 in acute aortic dissection, 3.09 ± 0.98 in chronic dissection, 3.16 ± 0.51 in thoracic aortic aneurysm and 4.58 ± 1.04 in acute myocardial ischemia, p < 0.05). Higher levels of MMP-1 and MMP-3 were detected on males. There was a positive correlation with increasing age (r = 0.38, p < 0.05). In patients operated for acute type A aortic dissection, the levels of MMP-1, MMP-3 and MMP-9 increased immediately after surgery, while the levels of MMP-2 decrease. At 24 hours postoperatively, levels of MMP -1, -2 and -9 are almost equal to the preoperative ones.

**Conclusion:**

Measurement of serum MMP levels in thoracic aortic disease and acute myocardial ischemia is a simple and relatively rapid laboratory test that could be used as a biochemical indicator of aortic disease or acute myocardial ischemia, when evaluated in combination with imaging techniques.

## Background

Matrix metalloproteinases (MMPs) constitute a large family of proteolytic enzymes containing a metal in their organic structure playing key roles in degradation of proteins in extracellular matrix and in tissue remodeling through complicated biological procedures [1-3]. This double action has been proven to be involved in the pathology of serious cardiovascular diseases, such as aortic aneurysm, dissection and coronary artery disease, which constitute the most common cause of death in developed countries [4-6].

In all the above pathological processes, but mostly in acute aortic dissection, a complex process is initiated for the repair and remodeling of the involved aortic wall. This process includes thrombus degradation through fibrinolytic activity and proteolysis of the extracellular matrix [7-9]. MMPs are proteolytic enzymes, specifically endopeptidases, whose catalytic mechanism involves a metal ion such as zinc (Zn^2+^) and calcium (Ca^2+^) [10]. Metalloproteinases, also called matrixins, include a large family of proteolytic enzymes, known as metzincin super-family. Their catalytic action is the degradation, mainly in neutral pH environment, of all proteins of the extracellular matrix [11]. In addition, they modulate many bioactive molecules at the cell surface and can act in concept to influence cell behaviour such as angiogenesis, migration, reproduction and immune system activity [12].

A number of metalloproteinases have been identified in blood serum that are categorized mainly in four groups: a) collagenases (MMP-1, MMP-8, MMP-13, MMP-18), b) gelatinases (MMP-2, MMP-9) c) stromelysins (MMP-3, MMP-10) and d) membrane-bound metalloproteinases (MMP-14, MMP-15, MMP-16, MMP-17, MMP-24, MMP-25) [13].

The aim of our study is to evaluate the levels of serum MMP-1, -2, -3 and -9 in acute and chronic aortic dissection, thoracic aortic aneurysm and acute myocardial ischemia compared to normal individuals and assess their clinical significance.

## Methods

A total of 80 consecutive patients managed in a single institution were prospectively included in the study over a period of two years. Patients were classified according to the underlying disease process in four groups: Group A consisted of 31 patients with acute aortic dissection, group B consisted of 18 patients with chronic aortic dissection, group C included 18 patients with thoracic aortic aneurysm and group D included 13 patients with acute coronary syndrome presenting with electrocardiographic changes indicative of myocardial ischemia with or without elevation of myocardial enzymes. Control group (Group E) consisted of 15 healthy individuals. Demographic data of the study population are presented in Table 1.

**Table 1:** Demographic characteristics of the study groups

**Characteristic**	**Group A** **(n = 31)**	**Group B** **(n = 18)**	**Group C** **(n = 18)**	**Group D** **(n = 13)**	**Group E** **(n = 15)**
Age mean ± SD	60.1 ± 14.8	60.6 ± 13.1	60.7 ± 13.7	65.4 ± 10.3	40.1 ± 5.2
Male/Female	26/8	17/1	14/4	13/0	3/12
Arterial hypertension (%)	22 (64.7%)	13 (72.2%)	15 (83.3%)	12 (92.3%)	0
Diabetes (%)	13 (38.2%)	3 (16.6%)	2 (11.1%)	4 (30.7%)	0
CAD (%)	11 (32.3%)	2 (11.1%)	8 (44.4%)	13 (100%)	0
Hyperlipidemia (%)	9 (26.4%)	7 (38.8%)	10 (55.5%)	9 (69.2%)	0
Chest pain (%)	33(97%)	0	1 (5.5%)	13 (100%)	0
ST-elevation (%)	6 (17.6%)	0	0	13 (100%)	0
Creatinine > 140 μmol/l (%)	8 (23.5%)	3 (16.6%)	2 (11.1)	2 (15.3%)	0

CAD: Coronary Artery Disease

Exclusion criteria included the presence of abdominal aortic aneurysm, use of corticosteroid or non-steroid anti-inflammatory medication, or a history of chronic pulmonary disease or malignancy of any type. All these clinical conditions have been associated with elevated levels of serum MMPs [5,11].

The following MMPs were measured in the blood serum of all individuals: MMP-1 (interstitial collagenase) as representative of collagenases, MMP-3 (stromelysin 1) as representative of stromelysins, MMP-2 (gelatinase A) and MMP-9 (gelatinase B) as representatives of gelatinases. Blood samples in group A were collected after diagnosis of acute aortic dissection was established mainly with computed tomography angiography and before any surgical intervention in case of Stanford A dissection. Seventeen patients in this group (50%), who were diagnosed with a Stanford A dissection, were operated for aortic root replacement. In these patients serial MMP serum levels were also calculated just after transfer of the patient from theater to ICU and at 12 and 24 hours postoperatively. Blood samples in groups B and C were collected before any surgical intervention (open or endovascular). Blood samples in group D were collected at the onset of acute thoracic pain.

All blood samples were collected through a central venous or peripheral catheter and centrifuged at 4,000 rpm. Then, the centrifuged serum samples were refrigerated at -27°C and stored until final analysis. Serum MMP levels were measured by using a quantitative sandwich enzyme-linked immunosorbent assay (ELISA) test (R&D Systems Europe, Abingdon, UK) according to the manufacturer's guidelines. All samples were measured as duplicates. The mean was calculated for data analysis. The levels of MMPs were determined according to a known curve which takes into account four parameters based on the following equation: 4 parameter- (y = (A-D)/(1+(x/C)^B)+D) and the use of 7 values as standards for the calculation of the standard curve. All MMPs values were measured as ng/ml.

## Statistical analysis

All data are presented as mean ± standard error. Quantitative variables were tested for normality of their distribution by the Kolmogorov-Smirnov test. For non-parametric quantitative variables, data were subjected to Kruskal-Wallis analysis and tested with Mann-Whitney U test, while for qualitative variables the chi-square test was used. The Spearman's test was used for regression analysis. Analyses were performed using the SPSS statistical package (version 11.5 for Windows). Differences were considered significant if p values were lower than 0.05.

## Results

**Figure 1:**
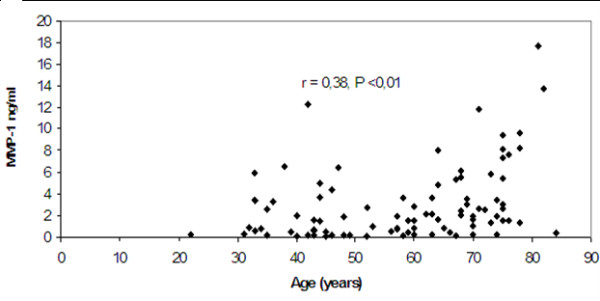
**Correlation of MMP-1 levels with increasing age**.

The mean values of the four studied metalloproteinases (MMP-1, MMP-2, MMP-3, MMP-9) in each group are presented in Table 2. It was observed that levels of MMP-1 and MMP-3 in all groups of patients were significantly higher (p < 0.05) in men compared to those found in women (Table 3). In addition, levels of MMP-1 showed a statistically significant positive correlation with age (Pearson correlation coefficient r = 0.38, p < 0.01, Figure 1).

**Table 2:** The levels of metalloproteinases (ng/ml) in studied groups of patients and healthy individuals

**MMPs**	**Disease**	**No. patients**	**Mean value ± St. error** **(ng/ml)**
MMP-1	Acute aortic dissection	31	2.97 ± 0.68
	Chronic aortic dissection	18	3.09 ± 0.98
	Aneurysm	18	3.16 ± 0.51
	Myocardial ischemia	13	4.58 ± 1.04
	Healthy individuals	15	1.10 ± 0.38
	Total	95	2.95 ± 0.34
			
MMP-2	Acute aortic dissection	31	151.62 ± 6.82
	Chronic aortic dissection	18	172.12 ± 11.79
	Aneurysm	18	156.64 ± 9.16
	Myocardial ischemia	13	180.94 ± 15.49
	Healthy individuals	15	171.37 ± 11.39
	Total	95	163.59 ± 4.58
			
MMP-3	Acute aortic dissection	31	12.92 ± 1.01
	Chronic aortic dissection	18	17.50 ± 3.60
	Aneurysm	18	17.64 ± 3.64
	Myocardial ischemia	13	17.33 ± 2.03
	Healthy individuals	15	10.66 ± 1.77
	Total	95	14.93 ± 1.11
			
MMP-9	Acute aortic dissection	31	412.93 ± 89.85
	Chronic aortic dissection	18	469.86 ± 56.57
	Aneurysm	18	342.97 ± 74.66
	Myocardial ischemia	13	409.74 ± 83.35
	Healthy individuals	15	400.13 ± 77.90
	Total	95	408.00 ± 37.78

**Table 3:** Comparison of the levels (ng/ml) of metalloproteinases in all groups according to sex

**Metalloproteinase**	**Sex of patient**	**No. patients**	**Mean value ± St. error** **(ng/ml)**	**p-value**
MMP-1 (ng/ml)	Men	70	3.44 ± 0.45	0.049
	Women	25	1.60 ± 0.29	
MMP-2 (ng/ml)	Men	70	165.60 ± 55.64	0.478
	Women	25	157.80 ± 17.38	
MMP-3 (ng/ml)	Men	70	17.40 ± 1.38	0.000
	Women	25	8.03 ± 0.58	
MMP-9 (ng/ml)	Men	70	437.53 ± 47.40	0.193
	Women	25	325.33 ± 52.69	

**Figure 2:**
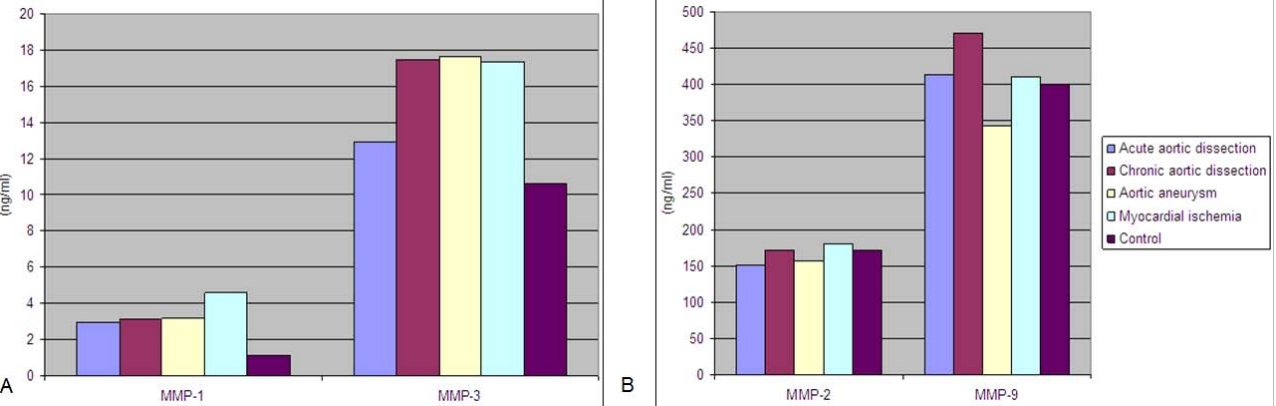
**A. Mean values of MMP-1 and -3 B. Mean values of MMP -2, and -9 in all study groups**.

Mean values of MMP-1 were lower in healthy individuals (group E) and higher in patients with acute myocardial ischemia (group D). Mean values of MMP-2 were lower in patients with acute aortic dissection (group A) and higher in patients with acute myocardial ischemia (group D). Mean values of MMP-3 were lower in the control group (group E) and higher in patients with thoracic aortic aneurysm (group C). As far as the mean values of MMP-9 are concerned, these were higher in patients with chronic aortic dissection (group B) and lower in patients with thoracic aortic aneurysm (group C) (Figure 2A, B).

MMP-1 serum levels in healthy individuals (group E) were significantly lower when compared to those observed in all other groups (p < 0.05). MMP-3 serum levels in healthy individuals (group E) were also found to be lower compared to the other groups, however this difference did not reach statistical significance. As far as the levels of MMP-2 and MMP-9 are concerned, serum levels were similar between groups.

Furthermore, blood serum levels of MMP-3 in patients with acute aortic dissection were significantly lower compared to those found in patients with acute myocardial ischemia (12.92 ± 1.10 versus 17.33 ± 2.03 ng/ml, p < 0.05). Mean values of MMP-1, MMP-2 and MMP-9 did not show significant difference between groups A and D. In addition, levels of MMP-1, -2, -3, -9 were not statistically different in patients with chronic aortic dissection (group B), aortic aneurysm (group C) and healthy individuals (group E).

Mean values of all MMPs showed a characteristic pattern of change in patients operated for acute type A aortic dissection. (Table 4). More specifically, the levels of MMP-1 increased immediately after the operation and decreased gradually at 12 and 24 hours thereafter. Levels of MMP-2 gradually decreased after the intervention and 12 hours afterwards, however they showed an increase at 24 hours postoperatively. Levels of MMP-3 were slightly increased after surgical intervention, while a more pronounced increase was observed at 12 and 24 hours after the intervention. Finally, levels of MMP-9 showed a peak immediately after the operation, while they decreased at 12 hours and then slightly increased at 24 hours.

**Table 4:** Variability in acute aortic dissection of the levels (ng/ml) of metalloproteinases before the surgical intervention, immediately after, at 12 and 24 hours after the intervention

**Metalloproteinase**	**Before surgical intervention**	**Immediately after**	**12 hours post-op**	**24 hours post-op**	**p-value**
MMP-1 (ng/ml)	3.73 ± 0.90	8.92 ± 3.92	6.62 ± 1.52	3.38 ± 1.25	0.010
MMP-2 (ng/ml)	152.61 ± 9.92	132.72 ± 5.18	117.23 ± 7.14	134.02 ± 8.06	0.015
MMP-3 (ng/ml)	12.39 ± 1.34	13.52 ± 1.14	23.71 ± 4.46	29.50 ± 6.25	0.00004
MMP-9 (ng/ml)	408.16 ± 142.57	616.65 ± 70.35	120.96 ± 19.15	123.36 ± 33.93	0.00001

Values represent means ± St. error

## Discussion

Matrix metalloproteinases play a significant role in the pathogenesis of aortic disease. The present study evaluated the levels of serum MMPs- 1, -2, -3 and -9 in patients with diseases of the thoracic aorta. These were compared with values obtained from patients with acute myocardial ischemia and from normal individuals.

The levels of MMP-1 were found to be lower in healthy individuals compared to patients with acute and chronic aortic dissection, aortic aneurysm and myocardial ischemia. Atherosclerosis is the main common pathologic finding in these disorders. Clinical studies have investigated the role of MMP-1 in this pathologic process [6,14,15]. These studies evaluated the levels of MMP-1 in the vessel's wall and not in blood serum as performed in the present study.

Kai et al. measured the levels of MMPs-2 and -9 in the blood serum of patients with acute myocardial ischemia and they revealed a significant increase compared to healthy individuals [16]. This is in contradiction with the results of the present study. Nambi et al. investigated the levels of MMP-1 in patients with coronary artery disease [17]. They have not found any difference and concluded that MMP-1 can not be used as a prognostic biomarker for coronary artery disease. On the contrary, we observed that mean values of MMP-1 in patients with acute myocardial ischemia were significantly higher compared to the control group. This difference can be attributed to the fact that patients in our study suffered from acute myocardial ischemia, and not chronic stable coronary artery disease. In addition, healthy individuals in the present study were of younger age which may have affected their MMP-1 levels.

MMP-1 levels in our study showed a positive correlation with age, a finding that has not been previously reported in the literature. In addition, the levels of MMP-1 were found to be significantly higher in men compared to women in all study groups. To the best of our knowledge, gender variability of MMP-1 has not been previously investigated in the literature.

As far as MMP-3 is concerned, it was found that the MMP-3 levels in patients with acute myocardial ischemia were significantly higher than those found in patients with acute aortic dissection. In many occasions patients with acute aortic dissection present with clinical and electrocardiographic signs that mimic myocardial ischemia, which make differential diagnosis difficult [18]. Numerous studies have reported high levels of MMP-3 in atherosclerotic plaques in any kind of aortic aneurysm [15,19,20]. High levels of MMP-3 have also been reported in the blood serum of patients with coronary arteries aneurysm [21]. MMP-3, as well as MMP-9, has been implicated in the pathogenesis and treatment of abdominal aortic aneurysms [22,23]. In a recent study by Monaco et al. it was found that the levels of MMP-3 in the blood serum decreased after endovascular repair of descending thoracic aortic aneurysms [24]. In addition, our study found that MMP-3, as also shown with MMP-1 levels, is increased in the blood serum of men compared to women in all groups.

The present study also investigated the pattern of change in the levels of the four metalloproteinases after surgical intervention in patients operated for acute aortic dissection. It was found that, immediately after the intervention, the levels of MMP-1, MMP-3 and MMP-9 are increased, while the levels of MMP-2 are decreased. At 24 hours postoperatively, levels of MMP -1, -2 and -9 are almost equal to the preoperative ones. Sangiorgi et al. found that after endovascular exclusion MMP-9 and MMP-3 levels decreased to a level similar to that of patients undergoing open repair [25]. The cardiopulmonary bypass has also been reported to affect serum levels of MMPs [26]. Therefore, duration of extracorporeal circulation is a factor that has to be considered when studying the levels of MMPs after surgical intervention.

As a conclusion, measurement of serum MMP levels in thoracic aortic disease and acute myocardial ischemia is a simple and relatively rapid laboratory test, which can be easily obtained even in the primary healthcare setting. Although increased MMP-1 and -3 levels are not specific and can not provide a definite clinical diagnosis, they can be used as a biochemical indicator of aortic disease or acute myocardial ischemia, when evaluated in combination with imaging techniques, such as computed tomography, magnetic resonance angiography or echocardiography. The concept of biochemical diagnosis of aortic dissection is an attractive option, as it is rapid, non-invasive, easy to perform and conceivably less costly than contemporary imaging studies. Further studies are required in order to draw precise conclusions on the clinical significance of MMP serum levels in diagnosis and monitoring of different therapeutic strategies in the management of cardiovascular diseases.

## Competing interests

The authors declare that they have no competing interests.

## Authors' contributions

GK carried out the design of the study, patient selection, collection of samples, performed analysis of the results and drafted the manuscript, PA performed the statistical analysis and drafted the manuscript, NC conceived of the study and participated in its design and coordination, CF contributed to the statistical analysis and drafted the manuscript, KA participated in patient selection and in study design and drafted the manuscript, ER contributed to patient selection and study design, HA participated in patient management and study design, KR and CP conceived of the study and participated in its design and coordination.
